# Current challenges and future of agricultural genomes to phenomes in the USA

**DOI:** 10.1186/s13059-023-03155-w

**Published:** 2024-01-03

**Authors:** Christopher K. Tuggle, Jennifer L. Clarke, Brenda M. Murdoch, Eric Lyons, Nicole M. Scott, Bedrich Beneš, Jacqueline D. Campbell, Henri Chung, Courtney L. Daigle, Sruti Das Choudhury, Jack C. M. Dekkers, Joao R. R. Dórea, David S. Ertl, Max Feldman, Breno O. Fragomeni, Janet E. Fulton, Carmela R. Guadagno, Darren E. Hagen, Andrew S. Hess, Luke M. Kramer, Carolyn J. Lawrence-Dill, Alexander E. Lipka, Thomas Lübberstedt, Fiona M. McCarthy, Stephanie D. McKay, Seth C. Murray, Penny K. Riggs, Troy N. Rowan, Moira J. Sheehan, Juan P. Steibel, Addie M. Thompson, Kara J. Thornton, Curtis P. Van Tassell, Patrick S. Schnable

**Affiliations:** 1 Iowa State University, Ames, IA USA; 2 University of Nebraska-Lincoln, Lincoln, NE USA; 3 University of Idaho, Moscow, ID USA; 4 University of Arizona, Tucson, AZ USA; 5 Purdue University, West Lafayette, IN USA; 6 USDA ARS, Ames, IA USA; 7 Texas A&M University, College Station, TX USA; 8 University of Wisconsin-Madison, Madison, WI USA; 9 Iowa Corn Growers Association, Johnston, USA; 10 USDA ARS, Wapato, WA USA; 11 University of Connecticut, Storrs, CT USA; 12 Hy-Line International, Dallas Center, IA USA; 13 University of Wyoming, Laramie, WY USA; 14 Oklahoma State University, Stillwater, OK USA; 15 University of Nevada-Reno, Reno, NV USA; 16 University of Illinois Urbana-Champaign, Champaign, IL USA; 17 University of Vermont, Burlington, VT USA; 18 University of Tennessee, Knoxville, TN USA; 19 Cornell University, Ithaca, NY USA; 20 Michigan State University, East Lansing, MI USA; 21 Utah State University, Logan, UT USA; 22 USDA ARS, Beltsville, MD USA

## Abstract

Dramatic improvements in measuring genetic variation across agriculturally relevant populations (genomics) must be matched by improvements in identifying and measuring relevant trait variation in such populations across many environments (phenomics). Identifying the most critical opportunities and challenges in genome to phenome (G2P) research is the focus of this paper. Previously (Genome Biol, 23(1):1–11, 2022), we laid out how Agricultural Genome to Phenome Initiative (AG2PI) will coordinate activities with USA federal government agencies expand public–private partnerships, and engage with external stakeholders to achieve a shared vision of future the AG2PI. Acting on this latter step, AG2PI organized the “Thinking Big: Visualizing the Future of AG2PI” two-day workshop held September 9–10, 2022, in Ames, Iowa, co-hosted with the United State Department of Agriculture’s National Institute of Food and Agriculture (USDA NIFA). During the meeting, attendees were asked to use their experience and curiosity to review the current status of agricultural genome to phenome (AG2P) work and envision the future of the AG2P field. The topic summaries composing this paper are distilled from two 1.5-h small group discussions. Challenges and solutions identified across multiple topics at the workshop were explored. We end our discussion with a vision for the future of agricultural progress, identifying two areas of innovation needed: (1) innovate in genetic improvement methods development and evaluation and (2) innovate in agricultural research processes to solve societal problems. To address these needs, we then provide six specific goals that we recommend be implemented immediately in support of advancing AG2P research.

**Supplementary Information:**

The online version contains supplementary material available at 10.1186/s13059-023-03155-w.

## Needs in agricultural production for genomes to phenomes

 The acceleration of population growth has increased the demands on agricultural systems and such demands have primarily been met in the last 50 years with increased production output through both genetic improvement and management [1]. It is predicted that the world population will exceed ten billion by 2050 [2], which will require more food to be produced with fewer resources. Due to the continuing expansion of the human population and changing consumer needs, current agricultural annual gains in production will need to be further enhanced to meet the challenges of decreasing land available for agricultural production, an increased need for sustainable production of nutritious food, feed, and fiber, and with the further challenge of addressing ethical and social issues concerning food production [3]. This necessary increase in production must not be associated with an increase in land or resource use but should capitalize on genetic potential to harness more efficient productivity in different and changing environments. Changes to farming environments associated with increases in extreme climate events and changes in land or water availability create additional challenges to ensure the stability of food production.

We propose that agriculture's production challenges can be tackled by an effective program that harnesses technological advances to better understand the genomes of agricultural species with the aim of developing novel management and modeling tools for improved predictions and, therefore, selection of superior individuals or cultivars in genetic populations [4]. Understanding “genome to phenome” (G2P) is a grand challenge for biology and is key to increasing the genetic improvement of agricultural resources [5]. Due to technological advancements and governmental investment in genome sequencing for all major crop and livestock species, collecting highly detailed genotypic information is now routine. Due to its complexity, phenotypic measurements are much less advanced. However, accurate and/or high-throughput phenotypic information on a large scale is crucial to both basic and applied sciences [6]. These phenotypes include both physiological/anatomical measures on individuals but also environmental parameters, as understanding the interaction between genotype and environment is important. In this context, molecular phenotypes, including epigenetic measures, will be an important component of understanding the biology of this interaction [7, 8]. Such phenotypic information on plants and animals, when combined with genomic information, enables researchers to (a) identify underlying molecular mechanisms of biological phenomena, (b) maintain or increase the genetic gain from selection in breeding programs, and (c) assess and optimize management strategies in production settings. Hence dramatic improvements in measuring genetic variation across populations (genomics) must be matched by improvements in identifying and measuring relevant trait variation in such populations across many environments (phenomics) to enable an understanding of the resultant mechanistic relationships. Genomic selection tools have resulted in considerable improvements for agriculturally important species [9, 10] but can only be achieved for traits that can be directly measured (because they are clearly defined), modeled, or are highly correlated with well-characterized traits. To remain competitive in the global market, agricultural production systems must develop new technology to not only improve systems to collect data across diverse environments, but also develop predictive analytics to best analyze such data. Improvement of these tools will allow researchers to be more innovative and successful in predicting important outcomes.

While predicting biological outcomes is complex, the use of very large datasets that cover deep phenotyping across many individuals has shown promise in predictive biology and medicine [11–14]. Thus, the agricultural community must find ways to enhance the creation and use of such data to address the efficient production of food and energy for our growing population, especially in the face of climate change. Over the last 50 years, more variation and less predictability in weather patterns have been observed which results in decreased availability and quality of agricultural products [15].

Against the backdrop of needing to increase production in an efficient and ecologically sound manner, food production will need to adapt to address novel environmental stressors through the production of more resilient crops and livestock. An increase in research capabilities will be crucial for successfully improving resilience of agricultural species; however, these must come not only from improvements in terms of methods to reliably measure genetic diversity in agricultural species, but also in terms of inclusion of the human groups involved in these efforts, especially those historically underrepresented or marginalized. To this end, diversity should be further expanded upon by increasing the breadth of training in subjects with historically low participation from agricultural-focused academic programs, including quantitative, molecular, and computational methods that are crucial for the predictive biology described above. This increase in diversity should also apply to the range of disciplines that are engaged in improving food production, economics, and consumer behavior, especially the social sciences.

Although these trends are global issues, we feel a direct impact in the USA. While every effort should be made to work with all collaborative countries and cultures on developing these needed improvements, the USA is poised to be a leader in developing a successful agricultural genome to phenome (AG2P) program. Through the gains in scientific capabilities and diversity of thought and production practices — and the subsequent improvement of agriculturally relevant products (i.e., species, breeds, and cultivars) — we will be able to produce populations that are more resilient to environmental change to meet the demands of future consumers. Foundational scientific knowledge funded by governmental agencies that leads to such improvements will be open and available to all researchers worldwide.

## Survey selection of discussion topics most critical to furthering G2P science

The main goals of the Agricultural Genome to Phenome Initiative (AG2PI; 1) are to strengthen the ties between and among G2P research communities and create a shared vision for G2P research where common problems can be solved together. AG2PI is a federally funded program in the USA that has global membership from 172 countries, including advisory members from outside the USA. In the spring of 2022, AG2PI asked members of its scientific advisory board and steering committee to list what they “view as the most critical opportunities and challenges in the area of AG2P research.” This input was categorized and the following eight emerging themes, or “topics” (Table 1), were included in a subsequent community survey in which participants were asked to mark the three topics [16] most critical for future R&D funding and [1] the most difficult to achieve. The survey was created in Qualtrics and open from June to July 2022. Personalized links to the survey were sent to members of the AG2PI and Animal Genetics Mappers (AnGenMap) listservs while general links were sent to AG2PI stakeholder and partner organization lists (full list of these organizations available at https://www.ag2pi.org/institutional-involvement/). All responses were collected anonymously. In combination, these lists reached individuals working in plant and animal sciences, engineering, computer science, social science, and other fields relevant to AG2P research and policy. It also reached individuals working across various sectors including academia, industry, governmental organizations, and other private and non-governmental organizations. Overall, 148 survey participants provided responses to all 15 questions; 60% of participants reside in the United States. The majority of respondents work in academia (57%) or a government agency (23%); the remaining work at a non-profit or non-governmental agency (8%), a for-profit company (6%) or other organization (6%). This survey built off two previous community surveys conducted by AG2PI in 2020 and 2021 [7].

**Table 1:** Topic areas identified by the AG2PI Steering Committee and Scientific Advisory Board as being most critical to furthering AG2P science, in no particular order

*Topics*	*Examples provided for community survey*
*Phenotyping technology development*	In vivo/low-to-moderate throughput versus remote/high throughput measurement tools, integration of these
*Predictive analytics development*	Advancing big data tools, integrating statistics with machine learning techniques and artificial intelligence
*Democratizing access to technology*	Increase fluency in statistical/computational approaches, community investment in flexible software solutions, subsidize new tech testing
*Convergence science*	Facilitating collaborations across disciplines, integration of disciplinary approaches
*Standardizing research methods and tools*	Terminology, data collection, data storage and re-use, pipelines, maximize data interoperability
*Advancing genomic research*	Pangenomics, statistical models, and methods for fully leveraging low-pass sequencing data, data and support systems for predicting variant effect on phenotype
*Advancing plant and animal breeding*	Re-analyzing past selection programs with modern tools, Identifying areas for synergies, support for integrating genomic and phenomic data to optimize breeding decisions
*Diversifying engagement*	Learn from the approaches of indigenous and urban farmers, building translational validation studies broad enough for diverse producer types to participate

**Fig. 1:**
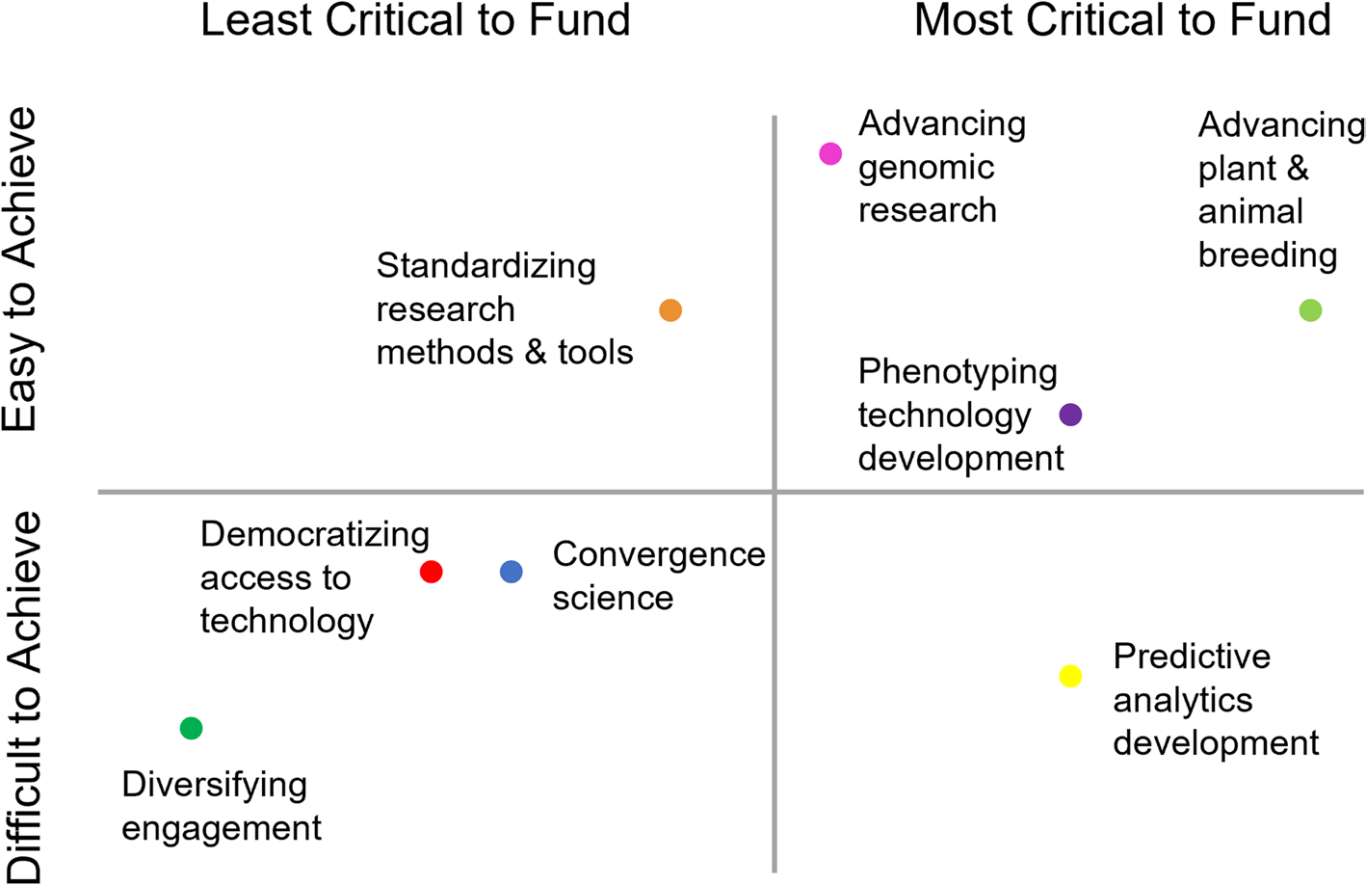
Survey rankings of topics for furthering AG2P science by both ease in achieving and criticality of funding

Results of the survey identified the intersections of which topics were viewed by the community as most critical for future research and development funding and easiest to achieve (the upper right quadrant of Fig. 1). An overview of these results was presented at the Thinking Big: Visualizing the Future of AG2PI” two-day workshop held September 9–10, 2022, in Ames, Iowa to prepare small group discussions among the workshop attendees. This workshop was a next step, as laid out in our previous paper [16], to advance AG2P goals and achieve a shared vision, including to coordinate activities with US federal government agencies including USDA, expand public–private partnerships, and engage with external stakeholders. Participants of this workshop included AG2PI co-PI/staff, seed grant recipients, advisory board members, stakeholder organization representatives, as well as USDA NIFA National Program Leaders and other thought leaders from the academia, commodity groups, and industry (Additional file 1: Appendix 1). Open text responses provided by the public survey-takers explaining why they selected topics as most critical to fund or most difficult to achieve also served as a launching point for small group discussions at the workshop. These discussions each explored one of the eight topics in terms of how it is a challenge in AG2P, what the costs are to society if the challenge is not met, possible solutions, and blue skies aspirations.

## Identified challenges and future steps by topic

In this section, each writing team summarized the discussions from the two 90-min small group sessions. Discussion groups included 6–8 participants, each representing a mix of crop researchers and livestock researchers, data scientists, social scientists, engineers, and other diverse stakeholders. After the first session, participants were requested to switch to another topic for the second period, to provide varied group dynamics and enrich the discussions. Detailed notes were taken by volunteer graduate students and postdoctoral researchers who also participated in the discussions. Those notes formed the basis for the following summaries. While there is some duplication in the needs and recommendations identified across topics, this is intentional as it highlights common problems and potential solutions. Several writing teams provided a full text of their summary, and these are included in Additional file 2: Appendix 2.

### Advancing plant and animal breeding

Increasing production without increasing land or resource use, coupled with changes in extreme climate events, presents new opportunities and challenges to breed animals and plants tailored to specific environments known to be more productive [17]. Animal and plant breeders have the ability to use genetic variation, develop tools, and deploy adapted cultivars and breeds to particular environments. Failing to advance methodologies could lead to inefficient land uses, shortages of agricultural products resulting in food insecurities, and cultivars and lines that are maladapted to their environments. Limited progress could result in competitive disadvantages, create dependencies, and endanger exports of agricultural goods. Efficiencies can be gained by coordinating public and private breeding efforts to minimize redundancies and increase complementarity. Similarly, several tools and methods that are species agnostic could be shared more widely. This will require intentional efforts to integrate plant and animal breeding groups. Exchange between species and kingdoms needs to be further improved, as well as interactions with other disciplines (e.g., medical genomics). To ultimately advance breeding into a more predictive and successful discipline, deeper functional understanding of traits needs to be obtained, both with regard to genes, environments, and genotype by environment interactions (GxE). Finally, education and extension professionals should be included to help educate consumers on breeding technologies for agricultural improvement. Integrative breeding would allow learning and innovation across species and kingdoms about theory, methods, and technologies.

### Phenotyping technology development

While past advances in phenotyping technologies focused on increasing throughput and reducing labor, new methods permit measurements that were previously impossible or impractical (e.g., growth curves, rumination, disease, and stress responses). Without innovative phenotyping technologies, researchers and producers will be unable to respond to shifting societal demands, particularly for poorly understood or complex consumer-driven traits such as texture, taste, or “sustainability” and the rate of genetic gain cannot be maintained or enhanced. Climate change compounds these challenges, as target environments continue to shift and existing agriculture genotypes have decreased output due to climate stresses [15]. New phenotyping technologies using cameras or wearable sensors (among others), provide a means to assess an individual's ability to cope with stressors in its environment. However, major challenges for new phenotyping technologies include ensuring the employed devices are resistant to the environment in which they are deployed (e.g., sunlight, dirt, moisture, or interactions with pen mates) and work in areas without reliable power or internet. Additionally, phenotyping equipment must be cost-effective, scalable, not impact the phenotypes to be measured, and easy to maintain. Data collected through these devices are often massive, and parallel advances in edge computing and data management are needed. Solutions to these challenges include attracting new talent and expertise to the field, increasing collaboration and teamwork across scientific domains (e.g., plant, animal, and computer scientists and engineers), and developing, testing, and benchmarking gold-standard datasets from new computational tools. Gold-standard datasets create their own challenge, however, as each area of AG2P will have different standards that will need to be defined (see also summary on Standardized Research Methods and Data). For example, the typical genotyping platform is very close to a gold standard (i.e., Illumina, Affymetrix, etc.) to generate reproducible genotype data that follows Mendelian inheritance. In livestock, coming up with a “gold standard” for individual phenotypes is a challenge. In crops, phenomes such as the shape of individual organs, color, and texture of the surface, can be measured, quantified, and stored in varying resolution and precision. The traits that are observed in most livestock species are mostly moderately heritable and not easily automatable, because animals are not stationary, their shape is fluid, and animals are housed in many different environments. It may take some creativity to achieve gold-standard phenomes in livestock.

Other solutions include creating public–private partnerships with appropriate intellectual property and data-sharing policies, with an emphasis on open-source standards for the global community of academic and industry scientists and researchers. Further advances in phenotyping technologies would lead to biosensors that are used on planters, sprayers, feed troughs, and plots, as well as deployed on animals and plants or in soil or digestive tracts to provide traceability, food security, and individualized nutritional management. In crops, these data and computational tools could create new production methods for determining the optimal distribution and spatial design of diversity in a field to maximize resilience rather than growing monocultures. In livestock, these can be used to capture behavioral or physiological data to identify animals that cope better with environmental stressors. Breeding could focus on optimization of main/rotation crops, co-evolution of rotation crops, livestock, and microbes, and interactions between forage and livestock.

### Advancing genomic (and epigenomic) research

Ensuring food security necessitates the advancement of genomic research, especially with regards to characterizing and integrating DNA sequence and structural variation with related central dogma–omic datasets (e.g., transcriptomic, epigenomic, proteomic, and metabolomic datasets). These data should encompass variation both within and across agronomically important species as well as their wild relatives. Open access to high-quality datasets has accelerated the refinement of genomic selection methodologies. However, additional data and integrative tools are necessary for genomic technologies to continue these improvements for agronomic outcomes. While genomic tools have become more accessible for many major agricultural species, most species’ genomes are represented by only a single cultivar or ecotype, which may not reflect commercial production varieties. Extensive sequencing of these selected individuals reduced the extent of discoverable genetic diversity and the consequences of this are now evident as we try to dissect the genetic architecture of economically important traits. To adequately utilize the genomic contribution from high-throughput phenotyping to novel trait information being obtained, more individuals, including wild progenitors, need to be sequenced at both genomic and epigenomic levels.

In addition, insufficient translation of advancements in basic genomic research to practical, usable analytical tools inhibits further germplasm improvement. Consequently, the full potential for genomic selection to accelerate genetic gain in agronomically important traits and species is unrealized [5, 18]. Additional scientific, funding, and human resources are needed to support young scientists and to help build a more cohesive agricultural genomics community. When public funding support for genomics does not keep pace with the costs of modern research, support staff resources decrease, and research-focused scientists must spend more time on non-science responsibilities, which impedes the workforce development of new scientists who often are highly innovative. Solutions to these challenges include using existing funding to efficiently generate high-resolution and more diverse –omics data, including spatiotemporal single-cell data, as well as computational tools to better integrate, visualize, and test biological hypotheses that are agnostic to the species (e.g., support both plants and animal research communities). Such resources can serve as reference datasets for the community to decrease data collection costs. Moreover, it is critical to engage scientists from industry to ensure relevance, and to ensure that data systems are accessible to the widest diversity of researchers, agriculture professionals, educators, and learners.

### Predictive analytics development

The general term “predictive analytics” encompasses a wide spectrum of data, processes, and techniques — including statistical, machine learning, and data mining methods — that attempt to uncover meaningful patterns (information) in data to permit accurate prediction of future events [19]. The goals of predictive analytics may include shrinking highly inbred crop populations to generate the most productive phenomes or select suitable parent populations for successive generations of livestock. This will require data from different or multiple temporal (days, weeks, or years) and spatial scales (individual plants/animals or entire ecosystems). The value of such predictive analytics to increase efficiency and decrease costs has been demonstrated in many fields [20–22].

To attain these goals, we, as an AG2P community, need to define data and software standards, create clear objectives, and share rigorously defined metrics of progress, as well as communicate and train users in best practices. The lack of existing gold-standard datasets and/or the sharing and access to such datasets has created testing and validation issues when newer technologies or datasets enter the field. Further, many individuals with training and experience in areas of agricultural science lack expertise in the fields of data science, machine learning, and artificial intelligence, while data scientists often lack a background in agriculture. This disparity often leads to gaps in common language and communication across and between researchers and educators in these fields, exacerbating existing disciplinary silos and slowing progress in identifying gold standards. Convergent, or transdisciplinary, team-building is required to overcome communication challenges, and, once overcome, these teams would benefit from a scientific culture that incentivizes collaborative science and trans-disciplinary interactions through promotion and rewards. This said, forward-looking, “big” ideas for solving current challenges in predictive analytics commonly focus on promoting bigger and more inclusive collaborative science. Prize competitions (e.g., Ansari X-Prize, NASA Solve) have been successful in bringing diverse individuals together to imagine and develop unique solutions, particularly those individuals who perceive the competition to be at the boundary of their expertise [23]. Additional ideas include hosting hackathons to develop, test, and identify the best predictor tools and software.

### Standardizing research methods and data

Standardizing research methods is a polarizing topic, especially in new research areas where new methods are being rapidly developed by disparate groups. Many researchers see there would be benefits to having standards but are concerned that this would stifle exploratory research or impose constraints on their work. Regardless, publicly funded research data and results must be Findable, Accessible, Interoperable, and Reusable (FAIR; [24]). These values are required for continued public confidence and support for such research efforts. Modern collaborative science will use public funds most efficiently and maintain high-quality value if all data are FAIR [25]. Examples of open collaborative science which incorporate these values in agriculture include Genomes to Fields (G2F, [26]) and Functional Annotation of Animal Genomics (FAANG, [27]); however, such projects are not routine. Given the historical and cultural impediments to sharing data and ideas across science and society, as a whole, an open science approach requires significant support to nurture the optimal AG2PI research enterprise. Data sharing is difficult as it requires both careful organization at the community level and individual adherence to recognized data standards. Early communication to avoid simply increasing the complexity of the problem is important, but as shown by the successful consortia listed above (G2F and FAANG), standardization can pay dividends.

Maintenance of FAIR data requires multiple levels of cognizance that impact individuals and groups (i.e., journal publication enforcement); however, it is crucial that such rules do not significantly impede scientific progress and data reporting itself. Supporting diversity in methods has many advantages, and it is helpful if those methods are FAIR and can be compared to gain a full appreciation of the advantages and disadvantages of various approaches. This is crucial and will require substantial communication across disparate communities with distinct expectations and protocols, including those outside AG2PI, such as AgBioData (www.agbiodata.org), NAPPN (www.plantphenotyping.org), the Research Data Alliance (https://www.rd-alliance.org), and FAANG (http://www.FAANG.org). Expanding current communication across these and similar groups should be encouraged. Specific topics for such discussions could include how to create long-term open data repositories, dedicated cloud-based analysis using standardized pipelines, and mechanisms to re-evaluate and periodically update such community resources. Visionary ideas such as artificial intelligence tools to find metadata automatically and potentially bring researchers working in overlapping areas together would be transformative.

### Democratizing access

Democratizing access to data and technology has been a focal point of the public and private sectors in data science-related disciplines. While this initiative is critical to disseminate knowledge and novel tools (e.g., code, software, and data), the scope of the democratization goes beyond data access through publicly available repositories. It requires other considerations to support and ensure that data generated is FAIR while appropriately acknowledging the studies, people or organizations that generated the data. Public dissemination of FAIR datasets provides researchers across the globe access to datasets that they may not have the infrastructure to generate or maintain but could use as well as contribute to. Aspects related to capacity building, such as access to computational resources, sensors, and digital literacy are other barriers that preclude access and research development [28]. Democratizing access to technology in agriculture would benefit small organizations by reducing the costs to develop new ideas, research projects, educational tools, and commercial products.

Education is a central component in democratizing access to data and technology in agriculture. A large portion of the agricultural community, which is composed of farmers, industry, cooperatives, and other associations, would benefit from training and support as well as user-friendly application tools that enable them to leverage data and technology development. The activities needed to democratize access to data and technology could be supported through federal funding agencies, where specific programs would finance research projects on the mechanisms to enhance data sharing and technology access in agriculture. These projects would also incentivize collaboration with other scientific domains, such as computer science, engineering, and social sciences, which is crucial to advance AG2P research quickly. Interdisciplinary work would create awareness of existing tools and strategies for best practices in data sharing. Strategies to monitor the benefits of democratizing access to data and technology are important to quickly identify weaknesses of sharing processes for timely adjustments and to justify future investments.

### Convergence science

The term convergence science is used to indicate a type of collaborative science that surpasses interdisciplinary and multidisciplinary science, requiring a unity of intellectual frameworks beyond the disciplinary perspective [29]. It has long been established that innovations and major breakthroughs come from convergent teams and from groups that are internally diverse [30, 31]. This convergence depends on the ability of the team members to build and sustain a productive collaborative environment—an ability that is both non-trivial and often neglected. A well-known example of the application of convergent science in biology is the Human Genome Project (HGP), which developed a multi-institutional, multi-national, and transdisciplinary team with a clearly defined, common goal to share responsibilities and benefits equitably within and beyond institutional and national boundaries [32]. The HGP fundamentally changed the way we do biology, transforming biology into an informational type of science and practically demonstrating transformative impacts of convergent science for technological advances and innovation. We note that fundamental scientific advances often leverage past discoveries, while the most complex questions remain unanswered [33]. This may be due to both intrinsic and extrinsic barriers. Intrinsic barriers exist mostly at the team-level and are based on practices and principles for developing strong, efficient, and long-lasting collaborative teams [34]. These include the lack of a common vernacular, forming an inclusive transdisciplinary team from the start (instead of ad hoc), reserving the time required for building trust, having a shared vision and clear expectations, and managing diverse viewpoints and personalities. Extrinsic barriers exist more at the institutional level and include the `silo’ mentality of expertise, incentivizing individual or independent research, and the lack of mindful development of convergent science teams (which build over longer time periods than most grant funding periods).

Funding agencies should be willing to devote resources to support this team building process as an initial step toward collaborative success in solving G2P. Immediate steps for developing convergent science can be divided into incentivizing creation of productive, transdisciplinary teams, and developing capacity to support convergent research. Examples of transdisciplinary team building are developing “go to” lists for a broad range of experts who are interested in addressing specific agricultural challenges, including stakeholders in team planning, and meeting mixers that support the development of transdisciplinary teams.

### Diversifying engagement

Agricultural stakeholders comprise a diversity of interests, ranging from commodity groups, individual producers, private corporations, environmental associations, government agencies, health organizations, and researchers. Several actions and Extension programs have successfully developed and implemented models of community engagement focused on identifying research priorities [35–37], co-production of science [38], and delivery of science-based recommendations to stakeholders and policymakers for over a century [39–41]. These programs have been very effective in reaching white rural stakeholders, but they have often failed in engaging minorities within these communities, such as individuals living in urban environments, indigenous people, and marginalized religious or ethnic communities [15, 42].

An important component to increasing the potential of G2P is to increase the diversity of scientists, stakeholders, and communities that are engaged in finding G2P solutions. There is scant literature on the impact of diversity on agricultural research; nevertheless, there are some reasonable assumptions we believe can be made. Communities that are poorly represented in scientific research could share — as well as gain — valuable knowledge and experience through increased opportunities and involvement in G2P research. Acceptance of diverse options to produce food increases when a broader producer community in involved [43, 44]. Further, increasing the diversity of consumer communities, including currently under-represented and indigenous groups, would increase overall marketable products by including a wider range of agricultural products. Forums like community listening sessions can be an effective way to learn from marginalized groups as participation is optional and engagement can be performed on the community’s terms [45]. Changing this deficit in engagement and connection will require substantial commitment, as there are historical and cultural impediments as well as a lack of budgetary prioritization for addressing this issue. This must be viewed as a long-term, complex problem that requires a sustained effort and will be most successful when viewed as a mutual benefit rather than one-sided. Specific ideas related to increasing diversity in G2P research include bringing G2P science into under-served communities, including workshops and hands-on demonstrations that are optimally related to community-specific resources and issues and that can spark interest in science and solve practical problems.

### Identifying solutions to challenges shared across topics

**Fig. 2:**
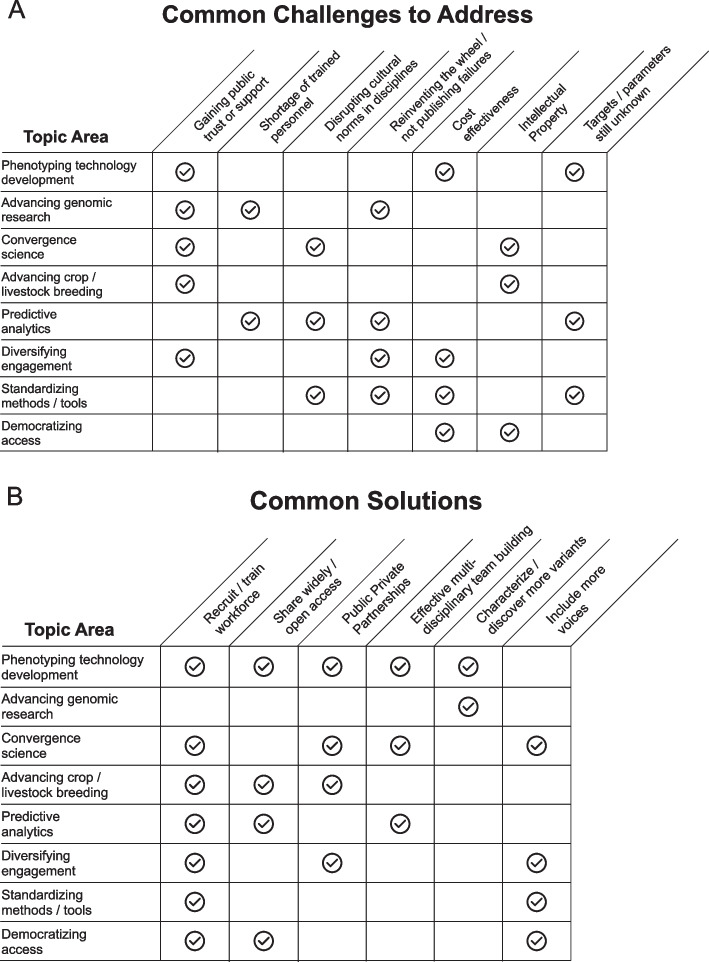
Challenges (**A**) and solutions (**B**) identified across the multiple topic discussions

Based on the above topic-specific discussions, several shared challenges as well as their solutions were identified (Fig. 2). First, an important challenge is identifying which yet-to-be-studied crop or livestock trait merits study, and then identifying what parameters to apply in such a study. For example, predictive analytics for genome to phenome could be advanced by initiating a single-cell genomics or genomics annotation project across a large number of species and integrating such data with other biological data types. The resulting data, covering multiple levels of analysis, would provide functional insight into cellular heterogeneity and provide new molecular traits for investigation; such a project could advance the underlying technology, identify data gaps, and further the methods for analyzing and managing the data. However, the technology that must be advanced to achieve this work may not be immediately cost-effective and the new avenues of study that are opened up would necessitate further financial support. To cut down on the financial input required to successfully meet these challenges, failed solutions would need to be shared, yet this does not generally occur. There is little incentive to publish failed attempts and so failures may be repeated, thereby reinventing the metaphorical wheel.

Another limiting factor to progress in linking phenomes with genomes is related to the lack of incentives to make data accessible or technology available when such data and technology may be expensive or proprietary, and when extra labor and funds—sometimes substantial – are necessary to ensure excellent quality standards in data and technology sharing [46]. Such efforts would go beyond just advancing the science faster but move closer to winning back public trust and support for research and its continued funding. Publishing datasets and code that follow FAIR principles require an up-front commitment and/or additional time from research teams and knowledge of the available open-science platforms for data sharing, primarily if large datasets are being published. Funding agencies are typically focused on supporting time-limited projects and cannot require research groups to maintain data resources in perpetuity; even national (National Center for Biotechnology Information, NCBI) and trans-national (European Bioinformatics Institute, EBI) public data warehouses cannot provide such resources for data from all biological research. Further, data availability requirements are different between journals, and once the data and associated materials are published, journals rely on researchers to dedicate time to answering questions and handling data retrieval complaints [47]. It will be necessary to break the cycle by disrupting the cultural norms that prop up these practices. Furthermore, intellectual property concerns hinder the public sharing of proprietary data, which further adds to the reinvention of the wheel and decreases the cost-effectiveness of public funding.

Solutions to this substantial problem of data sharing and accessibility will require institutional change; for example, journal policies that require method transparency and data sharing with appropriate metadata but can also develop from the ground up. As described below, improving the training of both data submitters and users is critical to change the expectations for data availability. As the agricultural community is quickly building large datasets that will require platforms with well-described policies to support decisions related to the lifetime of individual datasets, the development of an open and accessible research data infrastructure is crucial. This should include user-friendly interfaces to increase access to technology for a broader spectrum of users. For example, the development of technology based on mobile applications would make technology not only democratized and accessible but also scalable. Low- and middle-income countries have radically increased the use of mobile devices, going from 23 mobile subscriptions per 100 inhabitants in 2005 to 98.7/100 in 2017 [48–50]; therefore, the present is ripe with opportunity. However, while acceptance and participation in solutions by researchers will be important, only the funding agencies have the power to implement such needed changes in the research enterprise.

Addressing the challenges in agricultural G2P will require continuous support for trans-disciplinary science, starting from the very preliminary steps, e.g., assembling a research team, and throughout as team members develop productive communication strategies. This support is essential to overcome extrinsic barriers to convergent science, such as the silo mentality of expertise, the current emphasis of institutions on incentivizing individual or independent research, and differences in pay scales between fields of study and private and public sectors. Since building a convergent team is about building clear communication, clear expectations, and trust, this process will necessarily take time and resources, yet typical funding periods do not accommodate these timelines. Building effective teams can open novel opportunities to develop convergent science for agriculture by connecting extension experts, and their industry contacts, with researchers. Very often farmers and producers are interested in innovation but unable to take risks that may result in loss of production or profits. By building effective transdisciplinary teams we can overcome the institutional emphasis on conservative research that treads little new ground, and, instead, support a basic pillar of scientific research that knowledge is gained from unexpected outcomes in well-designed experiments. Further, if public–private partnerships focusing on real-world questions with sufficient data become more prevalent, subsequent incorporation of knowledge into better experiments and breeding protocols in the future is more likely. Efforts to address the legal and intellectual property constraints that vary across institutions and countries and that limit publicly funded research with industry proprietary breeds and germplasm (as well as access to metadata) would greatly improve the success of G2P research in agriculture.

Education and training are central components in common solutions for advancing genome to phenome research and applications (Fig. 2B). The use of complex technology for phenotyping will require a well-trained, astute workforce. Importantly, the curation of relevant platforms, best practices, and user awareness are key elements to overcoming data-sharing barriers in domains where these processes are not frequently routine. In general, graduate programs have not incorporated formal training on mechanisms for data sharing and technology democratization. Consequently, students and researchers are often unaware of the best-practices and benefits that data sharing would bring to these fields of research. Additionally, the rapid development of data and technologies that could be shared may overwhelm new users if not well organized with educational materials to inform data practitioners better. Developing workshops, webinars, and other hands-on activities will be important to train the next generation of students and researchers on data creation and sharing, including following standard protocols that will ensure access and reproducibility. An emphasis to support such training, together with providing the funding needed to describe and store these datasets, would synergize to improve data re-use, which can pay dividends to research funders in increased efficiency in knowledge output per input dollar.

Finally, understanding the needs of underrepresented stakeholders is a crucial objective for the long-term success of agricultural G2P research. The diversity that is inherent to the people, the disciplines, and the diverse heritage in agricultural products and processes can enrich G2P research. Failure to enhance research and investment in communities to ensure diversity, equity, inclusivity, and accessibility will not impart trust in the merits of scientific problem-solving and could contribute to the further marginalization or loss of minority groups and rural communities [18]. Lack of diverse engagement also stifles scientific creativity. The unique traditional knowledge held within underrepresented groups should be engaged to maximize finding diverse solutions to G2P prediction in the short term and can contribute to solving global problems in the long term [51, 52]. For example, examining technology and human interactions across all societal groups will help shape the future of work to increase opportunities for agriculture workers and productivity to create a community-driven workforce that reflects the diversity of the nation.

## Conclusions

The discussions summarized in this report provide insights on the challenges in genome to phenome research to meet the increased demands for agricultural production and sustainability. Based on these insights, specific recommendations for research investment are presented below, followed by critical milestones that, if achieved, will lead to a more inclusive agricultural community and benefits for producers and society in terms of sustainability, productivity, and profitability. Although the focus of the AG2PI program is advancement of research in the USA, these recommendations are applicable to the global research community. In fact, we welcome comments and exchange of ideas to build on these proposals.

### Innovate in genetic improvement methods development and evaluation

Without significant scientific innovations in AG2P, researchers and producers will be unable to respond to increasing and shifting societal demands for food, feed, and bioenergy. Selective breeding, including molecular markers, has proven powerful to improve many heritable traits [53–56], yet innovation is needed to address particularly complex and poorly understood traits, including those that are substantially affected by environment and or consumer-driven traits like food texture and taste. As consumer preferences change, new challenges arise in how to quantify and breed for new traits. Climate change compounds these challenges, as target environments continue to shift, and resources required for agricultural production become more constrained. Measuring new phenotypes that integrate environment and genetics, such as molecular epigenetic traits, will provide new tools, but there are significant unmet needs for agricultural producers focused on maximizing sustainability, productivity, efficiency, and profitability. We provide critical milestones below that highlight these needs in improving data analytics, enhancing public–private collaboration and data resources, and creating a workforce trained to exploit these new resources (bullets #1–4 below).

### Innovate in agricultural research processes to solve societal problems

Innovations and major breakthroughs can come from multi-disciplinary teams, and from highly diverse groups of people [30, 31]. This is particularly true for complex problems, such as food and energy production in the U.S., that have many, varied inputs and multiple measures of success. We must embrace the dedication required to develop such teams and provide the tools and resources to find genomic and phenomic answers to complex challenges to food, feed, and energy security. This development rests on building a more diverse, inclusive, and equitable research community through mechanisms such as improved and incentivized data and technology sharing, engaging with marginalized and underrepresented groups, and supporting the development and progress of effective and efficient scientific teams [18]. Several groups already exist that are committed to sharing and connecting agricultural data and research (i.e., AgBioData, CGIAR, CIMMYT, FAANG), and these and similar efforts could serve as the foundation for further development. In addition to the education and training mentioned already, significant investment in education and training in multi-disciplinary thinking and communication is also needed. Finally, we must encourage solutions that are species agnostic (i.e., that can be leveraged by multiple producer communities across plant and animal kingdoms). These needs, highlighted in bullets #5–6 below, will lead to a more cohesive agricultural community, one in which we can learn from each other and save time by solving common problems together.

We recommend that AG2PI funding be used to address the following critical goals:

1. Provide resources to address identified needs for G2P research, including the generation of benchmark testing datasets to identify success or failure and parameters to effectively advance from first- to second-generation AG2P predictive tools.
2. Remove current public–private barriers for collaborating with commercial entities that maintain large and relevant phenotypic datasets that are critical for the development and testing of predictive algorithms for agricultural G2P.
3. Establish comprehensive public genome/phenome knowledge bases that enable FAIR data sharing as a foundation for building on Federal investments for the exploration of biological function and the creation of new and improved agricultural products.
4. Increase progress toward developing and evaluating data analytics training programs and accelerate the training of scientists required for AG2P research and implementation, using curricula identified in AG2PI activities (e.g., field days, training workshops, and funded grants).
5. Expand the diversity of researchers, students and producers engaged in agricultural G2P activities through a sustained effort to bring G2P science and opportunities to underrepresented communities.
6. Identify additional gaps in knowledge, multidisciplinary team development, education/training, and analytical or quantitative methods relevant to AG2P (for example in genome function annotation, phenotyping methods, phenotype prediction, as well as convergence science), and strategize and initiate actions to fill those gaps.

### Supplementary Information

**Additional file 1: Appendix 1.** List of workshop attendees and their institutional affiliations.

**Additional file 2: Appendix 1.** Full text of writing team summaries.

**Additional file 3:** Review history.
