# Supplementary material for: Current challenges and future of agricultural genomes to phenomes in the USA
Source: Genome Biol. 2024 Jan 3;25:8. doi: 10.1186/s13059-023-03155-w (PMC10763150; doi:10.1186/s13059-023-03155-w)
Supplement: Supplementary file 1 — Additional file 1: Appendix 1. List of workshop attendees and their institutional affiliations. [file 13059_2023_3155_MOESM1_ESM.docx]

Appendix 1: List of workshop attendees and their institutional affiliations.

| **Name** | **Institution represented** |
| --- | --- |
| Christopher Tuggle | Agricultural Genome to Phenome Initiative |
| Jennifer Clarke | Agricultural Genome to Phenome Initiative |
| Brenda Murdoch | Agricultural Genome to Phenome Initiative |
| Eric Lyons | Agricultural Genome to Phenome Initiative |
| Nicole Scott | Agricultural Genome to Phenome Initiative |
| Jack Dekkers | Agricultural Genome to Phenome Initiative |
| David Ertl | Agricultural Genome to Phenome Initiative |
| Carolyn Lawrence-Dill | Agricultural Genome to Phenome Initiative |
| Patrick Schnable | Agricultural Genome to Phenome Initiative |
| Christian Tobias | USDA NIFA |
| Debora Hamernik | USDA NIFA |
| Angelica van Goor | USDA NIFA |
| James Reecy | Iowa State University |
| Juan Pedro Steibel | Iowa State University |
| Curt Van Tassel | USDA ARS |
| Fiona McCarthy | University of Arizona |
| Ben Rosen | USDA ARS |
| Alison Van Eenennaam | University of California -Davis |
| Darren Hagen | Oklahoma State University |
| Penny Riggs | Texas A&M University |
| Seth Murray | Texas A&M University |
| Nirav Merchant | CyVerse |
| Addie Thompson | Michigan State University / North American Plant Phenotyping Network |
| Alex Lipka | University of Illinois, Urbana-Champaign / NRSP-8 |
| Moira Sheehan | Breeding Insight |
| Thomas Lübberstedt | National Association of Plant Breeders |
| Jacqueline Campbell | AgBioData |
| Ellen Goddard | University of Alberta |
| Archie Clutter | University of Nebraska-Lincoln |
| Darrin Drollinger | ASABE |
| Ranveer Chandra | Microsoft Research |
| John Reiher | John Deere |
| Breno Fragomeni | University of Connecticut |
| Abby Stylianou | Saint Louis University |
| Sruti Das Choudhury | University of Nebraska-Lincoln |
| Carmela R. Guadagno | University of Wyoming |
| James Koltes | Iowa State University |
| Jinha Jung | Purdue University |
| Stephanie McKay | University of Vermont |
| Joao Dórea | University of Wisconsin |
| Andrew Hess | University of Nevada-Reno |
| Kara Thornton | Utah State University |
| Courtney Daigle | Texas A&M University |
| Troy Rowan | University of Tennessee-Knoxville |
| Bedrich Beneš | Purdue University |
| Max Feldman | USDA ARS |
| Kater Hake | Cotton Incorporated |
| Robyn Allscheid | National Corn Growers Association |
| Taylor Williamson | National Association of Wheat Growers |
| Fan-Li Chou | American Seed Trade Association |
| Dave Bubeck | American Seed Trade Association |
| Austin Putz | National Swine Improvement Federation |
| Janet Fulton | Poultry Breeders of America |
| Janet Lewis | CIMMYT |
| Ryan Corbett | Iowa State University |
| Katerina Holan | Iowa State University |
| Samantha Snodgrass | Iowa State University |
| Vishesh Bhatia | Iowa State University |
| Luke Kramer | Iowa State University |
| Henri Chung | Iowa State University |
| Tyler Foster | Iowa State University |
