# Supplementary material for: Current challenges and future of agricultural genomes to phenomes in the USA
Source: Genome Biol. 2024 Jan 3;25:8. doi: 10.1186/s13059-023-03155-w (PMC10763150; doi:10.1186/s13059-023-03155-w)
Supplement: Supplementary file 2 — Additional file 2: Appendix 1. Full text of writing team summaries. [file 13059_2023_3155_MOESM2_ESM.docx]

**Diversifying Engagement**

Agricultural stakeholders are a diverse group of people. A horizontal view of stakeholders includes a variety of commodity groups, individual farmers, private corporations, environmental associations, government agencies, health organizations, and researchers. A vertical view of the food supply chain shows that approximately 3% of the U.S. population (3 million people) is responsible for agricultural production, crop and animal protection, material processing, sale, and delivery, and virtually everyone relies upon agricultural goods, and food security (United States, 2017; United States, 2021). The racial distribution of American producers is severely skewed from the general US population showing that the current agricultural sector has not fully embraced a culture of Diversity, Equity, Inclusion, and Accessibility (DEIA) (**Figure 1**) (*https://www.nifa.usda.gov/diversity-equity-inclusion-accessibility-deia).*

Cooperative extension has transformed what was once an expert-driven, unilateral communication forum into a community-driven, train-the-trainer capacity-building model, with public conveners engaged in discourse on contemporary issues including renewable energy, workforce development, housing, and environmental change (French and Morse, 2015). Several actions and extension programs have successfully developed and implemented models of community engagement focused on identifying research priorities (Wang, 2014, James et al., 2014, Roche et al., 2015), co-production of science (Murray et al., 1999), and delivery of science-based recommendations to stakeholders and policymakers for over a century (Roche et al., 2013, O’Geen et al., 2015, Franz and Townson, 2008). These programs have been very effective in reaching white rural communities across the country, but they have often failed in engaging minorities within these communities, such as women and millennials living in urban environments, indigenous people, and marginalized religious or ethnic communities (Morse, 2009, Gowda et al., 2018).

One in five Americans lives in a food desert and many households are food insecure, with few to no convenient options for securing affordable fresh fruits and vegetables or other unprocessed foods. Food deserts often intersect with racial and structural inequities across the US with Hispanic, Black, and Native American communities being offered fewer options for healthy foods than in predominantly white areas with similar poverty rates. These communities have a cultural history of farming with innovative methods in local environments using culturally important specialty crops and cooking methods that underpin much of their community foundation. These foundations are at risk of crumbling permanently as local farms disappear due to the globalization of the food system.

Understanding the needs of underrepresented stakeholders and building cooperative teams capable of deploying science-based solutions that are community-driven while simultaneously investing in the agricultural sector from the bottom up instead of the top down is a crucial objective. Failure to enhance research and investment in communities to ensure DEIA will degrade trust in the merits of scientific problem-solving and will tragically contribute to the further marginalization of minority groups and the inevitable loss of numerous rural communities. Lack of diverse engagement also stifles scientific creativity. The unique perspectives and traditional knowledge possessed and utilized within rural communities and minority groups can be leveraged in facing global problems today and in the long term (Gilbert, 2021; Aquino, 2022). Looking at technology and human interactions across all societal groups will help shape the future of work to increase opportunities for agriculture workers and productivity to create a community-driven workforce that reflects the diversity of the nation. For instance, training students who return to serve their communities is an effective way to build competency and critical mass in these communities, while consciously avoiding a colonial approach to training, support, and education.

Unfortunately, there are many reasons why the research and extension community has struggled to build connections with indigenous and urban farmers or other stakeholders from marginalized communities. In many cases, engagement is just not happening. The lack of established connections and the need for the unique programming required to reach these groups deviates substantially from the strategies and content currently used to engage traditional audiences. Budget cuts to cooperative extension budgets in the 1990s and 2000s have forced extension programs to reduce programming and shift to a cost recovery model based on covering operational expenses through grants, fees, and partnerships with foundations or private corporations (French and Morse, 2015, Umali-Deininger, 1997). Community engagement from academics outside the extension community is not highly incentivized for career advancement. As a society, our lack of unprejudiced historical knowledge may in some cases manifest as a dearth of empathy toward marginalized groups. These issues are compounded by the fact that many individuals in these communities have faced unequal standards of existence, reduced opportunities for career advancement, and systematic deficiencies or outright abuse in the educational system. All these factors reduce our collective ability to trust each other and hamper thoughtful, empathetic communication, exchange of ideas, and joint problem-solving difficult. Ultimately, establishing better communication, listening to obtain a better understanding of challenges faced by these groups, and building trust between communities are needed for future engagement.

Effective listening and communication across all stakeholders is key to overcoming distrust, cultural differences, and even language that have made earnest efforts at DEIA difficult. Local extension offices can help in the develop effective outreach strategies and in some cases may be able to identify relevant potential contacts who are willing in joining community projects. Science road shows, workshops, and hands-on demonstrations are all events that help build community interest in scientific problem-solving. Demonstrations should spark curiosity by emphasizing the problem-solving nature of science and the excitement of discovery. It is important to understand what resources are scarce in many of these communities and that action will need tailored and accompanied by financial support and bi-directional mentorship. More equitable educational investment and enhanced holistic support is needed for participants from marginalized groups who interact with the university education system. People from disadvantaged backgrounds may not feel comfortable or need additional support in the university ecosystem due to differences in cultural norms, unfamiliarity with procedural details, and fewer resources relative to their more affluent peers. It is essential that community participants become part of the process early and throughout such that roadmaps can accommodate cultural norms and schedule events in the context of their home environment. Education focused on developing broadly applicable skills rather than simply training individuals solely to meet the common core standards would improve scientific literacy.

The desire for engagement differs between groups; some may be open to conversation whereas others may want to remain totally private. Trust building is a two-way street, as is effective listening others’ points of view. Engagement benefits when all parties recognize interaction as an opportunity for growth. These communities have much to teach the research community, and extension programs will fail unless we are truly present to learn. Forums like community listening sessions can be an effective way to learn from marginalized groups as participation is optional and engagement can be performed on the community’s terms (Bezahler, 2019). Performing background reading and research to learn about the community and its history prior to engagement can enhance sensitivity and gauge interest and impact. When appropriate, include other subject matter experts on the topics you will be discussing and deliver scientific content with excitement but in a way that is understandable to a general audience. Inclusion through the development of joint projects, where each entity contributes to project success or achievement is important. Ownership of positive outcomes is powerful for all communities.

Prioritizing DEIA must be a requirement for future actions at an institutional level. Too often the burden of implementing diversity and inclusion initiatives is left to those most severely affected by these issues. The effort needed and difficulty of engaging these communities needs to be better incentivized in academia, government, and private industry. The current working environment is placing emphasis on DEIA actions without fully understanding the complexity and time needed to culminate in successful efforts. Although the challenges are large, the outcomes are greatly beneficial to all parties. Inclusion, trust building, and knowledge sharing are keys to greater scientific understanding and building a more empathetic, sustainable world.

## References

Aquino L. 2022. Indigenous farmers reclaim time-honored techniques. In High Country News, issue Aug 30, 2022. <https://www.hcn.org/issues/54.9/indigenous-affairs-food-indigenous-farmers-reclaim-time-honored-techniques?utm_source=wcn1&utm_medium=email&utm_campaign=2022-08-30-Newsletter>

Bezahler A. 2019. 17 Indigenous Agricultural Programs to Recognize International Day of World’s Indigenous Peoples. In: FoodTank, issue Aug 17, 2019. <https://foodtank.com/news/2019/08/17-indigenous-agricultural-programs-to-recognize-international-day-of-worlds-indigenous-peoples/>

Gilbert S. 2021. Native Americans’ farming practices may help feed a warming world. In The Washington Post, issue Dec 10, 2021. <https://www.washingtonpost.com/climate-solutions/interactive/2021/native-americans-farming-practices-may-help-feed-warming-world/>

Gowda, P., J.L. Steiner, C. Olson, M. Boggess, T. Farrigan, and M.A. Grusak, 2018: Agriculture and Rural Communities. In *Impacts, Risks, and Adaptation in the United States: Fourth National Climate Assessment, Volume II* [Reidmiller, D.R., C.W. Avery, D.R. Easterling, K.E. Kunkel, K.L.M. Lewis, T.K. Maycock, and B.C. Stewart (eds.)]. U.S. Global Change Research Program, Washington, DC, USA, pp. 391–437. doi: 10.7930/NCA4.2018.CH10 <https://nca2018.globalchange.gov/chapter/10/>

Franz NK and Townson L. 2008. The nature of complex organizations: the case of

Cooperative Extension. In: Braverman MT, Engle M, Arnold ME, and Rennekamp RA (Eds). Program evaluation in a complex organizational system: lessons from Cooperative Extension. New Directions for Evaluation, number 120. San Francisco, CA: Jossey-Bass.

French C, and Morse G. (2015). Extension Stakeholder Engagement: An Exploration of Two Cases Exemplifying 21st Century Adaptions. Journal of Human Sciences and Extension, 3(2), 8.

James, Jeremy & Gornish, Elise & DiTomaso, Joseph & Davy, Josh & Doran, Morgan & Becchetti, Theresa & Lile, David & Brownsey, Philip & Laca, Emilio. (2015). Managing Medusahead (Taeniatherum caput-medusae) on Rangeland: A Meta-Analysis of Control Effects and Assessment of Stakeholder Needs. Rangeland Ecology & Management. 68.

Lincoln, N. K. (2019). Learning from indigenous agriculture. *Nature Sustainability*, *2*(3), 167-168.

Morse, GW. (Ed.). (2009). *The Minnesota response: Cooperative Extension’s money and mission crisis*. Bloomington, IN: *i*Universe.

Murray M, Cahn M, Caprile J, May D, Miyao G, Mullen B, Valencia J, and Weir B. 1999. University of California Cooperative Extension processing tomato cultivar evaluation program. HortTechnol 9: 36–39.

O'Geen, AT, & Saal, Matthew & Dahlke, Helen & Doll, David & Elkins, Rachel & Fulton, Allan & Fogg, Graham & Harter, Thomas & Hopmans, Jan & Ingels, Chuck & Niederholzer, Franz & Sandoval-Solis, Samuel & Verdegaal, Paul & Walkinshaw, Mike. (2015). Soil suitability index identifies potential areas for groundwater banking on agricultural lands. California Agriculture. 69. 75-84.

Roche LM, Kromschroeder L, Atwill ER, et al. 2013. Water quality conditions associated with cattle grazing and recreation on national forest lands. PLoSONE 8: e68127

Roche L, Schohr T, Derner J, Lubell M, Cutts B, Kachergis E, Eviner V, and Tate, K. (2015). Sustaining Working Rangelands: Insights from Rancher Decision Making. Rangeland Ecology & Management. 68.

Umali-Deininger, D. (1997). Public and private agricultural Extension: Partners or rivals? *The World Bank Research Observer*, *12*(2), 203–22

United States. (2021). U.S. Census Bureau: 2021 American Community Survey 1-Year Estimates. Web Archive. [https://data.census.gov/cedsci/table?q=Race&t=-00%3A001%3ARace and Ethnicity&tid=ACSSPP1Y2021.S0201](https://data.census.gov/cedsci/table?q=Race&t=-00%3A001%3ARace%20and%20Ethnicity&tid=ACSSPP1Y2021.S0201).

United States. (2017). U.S Census of Agriculture. United States, 2017. Web Archive. <https://www.nass.usda.gov/Publications/AgCensus/2017/>.

Wang, S.L. (2014). Cooperative Extension System: Trends and Economic Impacts on U.S. Agriculture. Choices: The Magazine of Food, Farm, and Resource. volume 29.

## Figures

**Figure 1**: Self-described racial demographics in the United States in **a**) the 3 million agricultural producers (United States, 2017) and **b**) the 330 million US residents (United States, 2021).

**Fig 1a**

**Fig 1b**

**Convergence Science**

The term convergent science - sometimes referred to as transdisciplinary science - is used to indicate a type of collaborative science that surpasses interdisciplinary and multidisciplinary science, requiring a unity of intellectual frameworks beyond the disciplinary perspective^1^. Grand societal challenges are highly complex, and they can only be solved by cross-disciplinary teams of researchers, hence the critically important role of convergent science. For example, agricultural production and food and feed security, both of which depend on advances in our understanding of genome-to-phenome, require solutions that can only come from the convergence of expertise from various disciplines. However, this convergence depends on the team members’ ability to build and sustain a productive collaborative environment - an ability that is both non-trivial and often neglected. The delivery of an efficient response to pressing societal needs will influence national and global growth resulting in long-term scientific, economic, and innovative sustainability. This will not be possible without the intersection of skills, resources, ideas, technologies, and expertise across different fields. It is now incumbent for the scientific community to develop and sustain an ethos of convergence to increase the efficiency of the actions targeting societal grand challenges while avoiding the waste of resources.

It has long been established that innovations and major breakthroughs come from multi-disciplinary teams, and from highly diverse groups of people^2,3^. We note that each generation solves the set of challenges that they are best able to address, leaving additional progress to the next generation; while fundamental scientific advances often leverage past discoveries, the most complex questions stay unanswered^4^. Agricultural advances are an excellent example of a challenging scientific landscape that it is unlikely can be solved by a uni-disciplinary team. The complexity of these challenges calls for transdisciplinary scientists to ensure that solutions proposed by experts in a particular field do not cause unintended impacts in another area (*e.g.*, the use of growth hormone in feedlots is biologically and economically sound but can negatively affect the environment). Unfortunately, the impact of these possible interferences is hard to measure as there may be direct economic or production losses but rather loss of public trust in the scientific community or delivery of products and technologies that are not feasible, efficient, or adopted by their intended users. A well-known example of the application of convergent science in biology is the Human Genome Project (HGP), which developed a multi-institutional, transdisciplinary team with a clearly defined, common goal to share benefits equitably within and beyond institutional boundaries^5^. While the exact return on investment for the HGP is disputed, it is in the order of $100-$140/ dollar invested^6^. More importantly, the HGP fundamentally changed the way we do biology, transforming biology into an informational type of science and practically demonstrating the transformative impact of convergent science for technological advances and innovation.

The barriers to using convergent science for AG2P can be broadly categorized as intrinsic or extrinsic. Intrinsic barriers are based on practices and principles for developing strong, efficient, and long-lasting collaborative teams^7^. These include the lack of a common vernacular, forming an inclusive transdisciplinary team from the start (instead of *ad hoc)*, the time required for building trust, having a shared vision and clear expectations, and managing diverse viewpoints and personalities. Related to these intrinsic barriers are tensions that may develop because of misaligned expectations relating to issues such as intellectual property, publications, data sharing and authorship. In many cases these intrinsic barriers can be overcome by careful planning and the inclusion of expertise in developing productive teams. Full convergence can only be achieved by reducing these barriers to forming integrated, trans-disciplinary teams, including embracing an inclusive view on expertise and consideration of team dynamics and expectations. Practically, this translates into continuous support for trans-disciplinary science from the very preliminary steps of assembling the team throughout the time required for team members to develop productive communication strategies. Extrinsic barriers tend to be more institutional and include the silo mentality of expertise, institutional barriers to collaboration such as missing guidelines for articulation agreements, incentivizing individual or independent research, differences in pay scales between fields of study and private and public sectors, the lack of mindful development of convergent science over selecting team members based upon familiar and longer timelines which do not align with typical funding periods. We note that some fields of expertise are more familiar with collaborative projects (*e.g*., the convergent approach of the HGP established a precedent for convergent genomics research) and that not all collaborative research is convergent (*e.g*., a cross-institutional, collaborative team may consist entirely of expertise from a single field of study). As they are rooted in established institutions, extrinsic barriers are more difficult to overcome. There are several examples of institutes, such as the Morgridge Institute in Wisconsin or the MIT Koch Institute (MA), set up specifically for convergent science and we note the examples of successful convergent science projects have all had separate, dedicated funding in addition to physical infrastructure.

Immediate steps for developing convergent science can be divided into promoting productive, transdisciplinary teams and developing capacity to support convergent research. Examples of transdisciplinary team building are developing “go to” lists for a broad range of experts interested in addressing agricultural challenges, including stakeholders in team planning and meeting mixers which support the development of transdisciplinary teams. We note that virtual and hybrid meetings provide new opportunities to attract people from different disciplines who may be interested in being part of a transdisciplinary team focused on a specific challenge. It is important to encourage team building exercises and expertise in the initial phases of developing convergent teams and to ensure communication strategies that enable clear communication across disciplines. Since building a convergent team is about building clear communication, clear expectations and trust, this process will necessarily take time and resources devoted to this team building and this process needs to be supported. Along with team building are opportunities to develop capacity by providing funds specifically for convergent science. Several funding agencies started recognizing the relevance of already integrated or transdisciplinary funding opportunities. The National Science Foundation Reports Growing Research Convergence as one of the Ten Big Ideas - the merging of approaches and technologies from widely diverse fields to stimulate innovation^8^.

Encouraging agencies to highlight these opportunities and related opportunities (*e.g*., conference awards to support meetings which develop transdisciplinary teams) is essential. Part of this may be seed grants focused on establishing convergent teams. Moreover, there are several convergent science teams in agriculture (*e.g*., Virtual Dairy Brain, NAPPN, Predictive Plant Phenomics, and AI Institute for Resilient Agriculture) which can serve as models for developing convergent science in agriculture. While there are some funding opportunities to support academia-industry partnerships, they are often very limited in both scope and opportunity. Broadening these opportunities and providing novel venues to develop projects with industry partners will require balancing expectations from both groups (for example, industry often reaches out with very focused, short-term projects in mind). One area where both groups may benefit is *via* student internships within industry, with the student expected to bring back skills to the graduate program. This example of developing a bi-directional flow of students, training and knowledge would require capacity development to support internships within programs and would be expected to provide feedback for programs which could support subsequent curriculum development to support workforce development. Along with student training are more opportunities for mini-courses/certificate programs for the agriculture workforce to gain multidisciplinary skills (*e.g.*, an animal science degree-holder learning data science/statistics) which are needed in the industries of tomorrow. Another area where there are novel opportunities to develop convergent science for agriculture is by linking in Extension expertise and their industry contacts with researchers. Very often farmers/producers are interested in innovation but unable to take risks which may result in loss of production or profits.

As noted at the beginning of this section, solving grand challenges facing agriculture will rely on bringing together expertise from many domains. Convergent science will be critical for addressing USDA Strategic Goals focused around supporting US agriculture including managing impacts due to climate change; ensuring the agricultural system is equitable, resilient, and prosperous and providing an equitable and competitive marketplace for all agricultural producers. Developing convergence science with opportunities for researchers to work with experts in social sciences, economics, and rural development (for example) will ensure that AG2PI research better addresses community needs and supports the inclusion of diverse groups in agriculture (*e.g*., urban farming, tribal groups). Moreover, expertise in communication and Extension will ensure that producers are informed of technical advances which may be relevant to them.

**Reference List:**

1. Roco MC. Principles of convergence in nature and society and their application: from nanoscale, digits, and logic steps to global progress. J Nanopart Res. 2020;22(11):321. doi: 10.1007/s11051-020-05032-0. Epub 2020 Oct 22. PMID: 33106748; PMCID: PMC7577848.
2. Kuhn: Kuhn, Thomas S. The structure of scientific revolutions. Vol. 111. University of Chicago Press: Chicago, 1970.
3. Hofstra, B., Kulkarni, V. V., Munoz-Najar Galvez, S., He, B., Jurafsky, D., & McFarland, D. A. (2020). The diversity–innovation paradox in science. Proceedings of the National Academy of Sciences, 117(17), 9284-9291. <https://doi.org/10.1073/pnas.191537811>
4. Dörner Dietrich, Funke Joachim. Complex Problem Solving: What It Is and What It Is Not

Frontiers in Psychology (2017), 8 DOI=10.3389/fpsyg.2017.01153

1. Petersen, Alexander M., et al. "Cross-disciplinary evolution of the genomics revolution." Science advances 4.8 (2018): eaat4211. PMID: 30116784.
2. Battelle Memorial Institute titled “Economic Impact of the Human Genome Project AND <https://www.genome.gov/27544383/calculating-the-economic-impact-of-the-human-genome-project>.
3. Tuckman, Bruce W (1965). "Developmental sequence in small groups". Psychological Bulletin. 63 (6): 384–399. doi:10.1037/h0022100. PMID 14314073.
4. <https://www.nsf.gov/news/special_reports/big_ideas/>

**Advancing Plant and Animal Breeding**

**Importance of challenge**

Due to a growing human population and changing consumer needs, the current annual genetic gains will need to be further enhanced, in order to meet the projected increase in global demand for plant and animal products. Increasing production must not be associated with increasing land or resource use, which pushes the challenge to productivity instead of quantity. The widening gap between needed and actual progress in genetic improvement is endangering the availability of sufficient quantity and quality of food, feed, and energy products. Varying environments associated with changes in extreme climate events, land, and water availability create additional challenges to ensure stability of food production. With this challenge, there is also a new opportunity to breed animals and plants tailored to specific environments. Animal and plant breeding have the genetic variation and ability to deploy and develop tools to adapt cultivars and breeds to particular environments. There is a need to move towards optimizing breeding within cropping or agricultural systems rather than individual species. Integrative breeding would allow learning and innovation across species and kingdoms about theory, methods and technologies.

An unknown risk is whether current increases in genetic gains can be sustained long-term, or if genetic diversity within species will plateau at some point. In addition, the necessity of increased production and productivity may result in higher inbreeding rates, which may negatively impact productivity, genetic variation, and overall health of the organisms involved in breeding programs.

**Impact on science if the challenge is not met**

Lack of progress in animal and plant breeding will slow down scientific progress in areas such as population and quantitative genetics, biostatistics, among other areas. Since genomics and similar technologies are mainstream, limited scientific progress in those areas may affect other disciplines and economic areas beyond animal and plant breeding. In addition to impacts on science, failing to advance methodologies in plant and animal breeding at a global level will lead to shortages in agricultural products where increased prices could lead to malnutrition and migration among the world’s most vulnerable populations. Limited progress at a national level would result in competitive disadvantages to other countries, create dependencies for services, and endanger exports of agricultural goods. Moreover, limited progress in developed countries can increase the disparity between developed and developing countries. Such disparities can result in social and economic problems both domestic and abroad. Failing to advance the breeding field may result in cultivars and lines that are maladapted to their environments which would create a genetic vulnerability in agriculture. Finally, delays in genetic progress would result in increased use of land, water, and resources creating a less sustainable agricultural system.

**Barriers that currently exist in meeting the challenge**

Causes for limited progress include insufficient funding and support, legal constraints, e.g., for genome editing, and exhausted genetic diversity in some agricultural species. More specifically, these were the main barriers, mentioned at the AG2PI workshop:

1. Financial support is insufficient. For example, U.S. animal breeding receives 4-20x less funding for major species compared to Europe (not including national funding within EU). Private plant breeding programs have a far greater budget for few major crops, than the funding available to public breeders in the U.S. for all species together.
2. Funding is usually short term (few years) in contrast to the needed long-term support of breeding programs and continuity.
3. Breeding faculty are trained to attract any funding from any source, which may come from unrelated areas. This diverts research efforts from actual breeding research and decreases program success rate. The time spent on grants that will not be funded may decrease the actual time dedicated to research.
4. Legal and IP constraints limit research on industry standard lines (e.g., genotyping of proprietary breeds and germplasm prohibited; limited access to metadata and publication results)
5. Limited technology acceptance (e.g. editing, GM), and scientific “illiteracy” in society
6. Inconsistent regulations between countries
7. Mismatch between technological status quo (e.g., progress in sequencing) and understanding causality.. There is a large research effort on associating variants to complex phenotypes. However, those studies are underappreciated by funding agencies, industry, and stakeholders. Very little effort is made to improve such methods, which further reduces enthusiasm in genomics research. Further, genetic prediction methodology must find ways to leverage causal and functional variation to increase selection accuracies.
8. Narrow funding calls limit diversity and innovation in research

**Actions to solve the challenge now**

With current funding available from USDA NIFA for animal and plant breeding programs, it would be desirable to allow for longer-term and more continuous research projects. For example, instead of supporting projects that end after 3-4 years, it should be possible to request one or more extensions, in case the initial project period of 3-4 years was successful. Efficiencies could be gained by coordinating public and private breeding efforts, minimizing redundancies, rather trying to be complementary. Similarly, several tools and methods are species agnostic and should be shared widely. This will require intentional efforts to integrate plant and animal breeding groups. Important roles of public in contrast to private breeding efforts include characterization of germplasm and genetic resources, and to make those better accessible for elite breeding programs. Another important role is workforce development. Both of these important roles are limited by the nature and quantity of funding.

**Short (S), medium (M) and long (L) term priorities, blue (B) skies ideas**

Specific groups or locations should serve as hubs or core laboratories for particular areas, or technologies (S). This could apply to particular phenotyping technologies, particular bioinformatic or database tasks, maintenance of cell lines, etc. Exchange between species and kingdoms needs to be further improved (M), as well as interactions with other disciplines (e.g., medical genomics). To ultimately develop breeding into a discipline comparable to engineering, deeper functional understanding of traits needs to be obtained (L), both with regard to genes, environments, and GxE. To be able to utilize this understanding fully, progress in biotechnologies such as reproductive biology is essential (B) – to for example be able to replace the germline of sires with that of elite sires. Finally, the general public has to be supportive of using breeding technologies for agricultural improvement, which can be accomplished by education and extension (L).

**Predictive Analytics Development**

*Advancing big data tools, integrating statistics with machine learning techniques and artificial intelligence, etc.*

**Background – The Challenge and Importance of Predictive Analytics:**

We have reached a point where data collecting has become ubiquitous. We continuously capture data about the environment, and advanced sensors allow for large-scale, high-precision, and real-time gathering of data about plants and animals. While much of this data can be captured in the wild, specialized phenotyping facilities allow capturing high-quality, controlled data. The term *predictive analytics* encompasses techniques, including statistics, machine learning, and data mining, that attempt to uncover meaningful patterns (information) in such data (Abbott, 2014). One of the key functions of predictive analytics is to inform our decisions using the best information possible to hopefully make the best decisions for the future. While the general term predictive analytics encompasses a wide spectrum of data and processes, the goals typically differ for plants and animal applications. In plants, the typical aim is to shrink populations to the most productive phenomes, while animal breeding schemes attempt to select parents for successive generations. Another unclear objective is the temporal (days, weeks, or years) and spatial scale (individual plants or the entire ecosystems) of the predictions.

An important challenge resulting from the wide variety of processes, data sources, and objectives in agriculture is lack of standards for data formats and software communication. While many scientific fields, such as computer vision, have clearly defined benchmarks and metrics of success, the variety of options in agriculture makes similar standards difficult to define. While our eventual goal is to predict phenome from the genome, the easily achievable goals are to detect patterns and causalities. We need to develop improved methods to analyze large datasets as the storage and processing improvements are not growing as quickly as the data are growing.

Another challenge is that of disciplinary silos. The traditional approach to attack predictive analysis involves researchers with divergent expertise, an expert in data analytics and another domain content expert. While it is usually easier to train a data analyst the subject matter needed, a new generation of transdisciplinary researchers will provide new insights into both disciplines.

**Potential Impact On Science If The Challenge Is Not Met:**

The scientific community must find ways to enhance predictive analytics to address many important issues that will impact every level of our growing population. It is widely believed that the world population will exceed ten billion by 2050. This increase in population will have a large impact on agriculture as more food will need to be produced with less resources (land, water, etc.). One of the biggest issues facing the entire population is climate change. Over the last 50 years, more variation and less predictability in weather patterns has been observed, and results in overall decreased availability and quality of agricultural products. Although these trends are global issues, there is also a direct impact on the US. For the US to remain competitive in the global market, agricultural production systems must develop new technology to improve predictive analytics. Improvement of these tools will allow for researchers to be more innovative and successful in predicting important outcomes.

We need to define data and software standards, clear objectives, and rigorously defined metrics of progress. We should create and maintain a list of accepted algorithmic solutions to these problems that would allow for a clear definition of new tasks. For example, one goal might be the full 3D reconstruction of plants with all geometric details. The task can be defined on various data (images, point clouds) and the precision can be determined as the deviation of the prediction model from certain manually measured phenotypes. New algorithms could be compared against these standards by comparing speed and precision of these approaches, and the more accurate prediction tools should also be able to reduce the cost of traditional genomic analyses.

**Barriers That Currently Exist:**

Existing barriers hinder advancements in predictive analytics. One such impediment that is instrumental to the future of research and development of predictive analytics is the extreme shortage of trained personnel. Training of students and scientists is a long-standing issue, as many individuals with training and experience in areas of agricultural science lack expertise in the fields of data science, machine learning, and artificial intelligence, while data scientists often lack a background in agriculture. This disparity often leads to a lack of a common language and communication across and between researchers and educators in these fields, exacerbating existing discipline silos.

Existing scientific challenges in predictive analytics are great. As increased complexity of a system introduces more chaos into potential outcomes, it is still unclear what characteristics within complex biological systems are predictable. The opportunity to reduce system complexity and narrow our lists of solutions relies on two primary gaps. 1) From a biological perspective, our current understanding of the biology underpinning traits of interests and on the impacts of a changing environment on those traits is lacking. 2) The lack of existing gold-standard datasets and/or the sharing and access to such datasets has created testing and validation issues when newer technologies or datasets enter the field.

A unique challenge that must be considered is the current culture of risk aversion by academic administration in science, thereby promoting “safe” research making incremental gains in knowledge rather than encouraging transformational investigations that may result in large leaps in understanding. This approach, seen as necessary for successful funding, publications, and career advancement, has created an overall lack of incentives promoting or rewarding collaborative science and trans-disciplinary interactions. Larger, collaborative efforts must be undertaken to mitigate costs of establishing such long-term training and research programs.

**Actions That Can Be Performed Now:**

Existing barriers and resolving these challenges may be addressed by implementing a number of short and long-term strategies. In the near-term, there is an urgent need to attract and train students in predictive analytics and we must expand our search and training into disciplines beyond plant and animal sciences. To address this need, we must identify areas of core competency for trainees in predictive analytics. Further, we must focus on reducing the urge to build and maintain discipline-specific silos in academia. Promoting cross-disciplinary interactions between data scientists, modelers, statisticians, and agricultural scientists is necessary to holistically identify current and forthcoming barriers or challenges in predictive analytics. The non-traditional partnerships are necessary to address the complexities associated with biotic and abiotic interactions in agriculture. Better understanding the biology of the organism and its interactions with the changing environment will reduce the complexity of the system in which predictions are made. Regarding near-term solutions to the science of predictive analytics in agriculture, we must identify existing datasets and code that have been quality verified and make these data and codes publicly available. To accomplish this, we may need to incentivize open sharing via accolades/awards for data sharing and reuse to properly acknowledge those who have generated and contributed to the field.

Long-term solutions in predictive analytics should include reducing the unintended encouragement of risk mitigation of scientific research and approach. Junior faculty are often directly or indirectly encouraged to engage in research that can be directly attributable to them, potentially reducing their participation in collaborative research efforts carrying out “big science”. Funding agencies and review panels request and reward research grant proposals with limited risk and high potential for successful outcomes. This behavior further promotes research projects viewed as safe and smaller in scope. Using an individual’s success in grantsmanship and publication by academic administrators during the promotion process further encourages risk aversion by scientists. Large, risky projects carried out for the sake of science are eschewed in favor of smaller projects that are more assured of generating publishable units. Funding agencies should encourage high risk, high reward research often best carried out by large, collaborative, transdisciplinary groups. Adjusting criteria used by academic administrators for promotion would encourage junior scientists to abandon discipline silos in favor of building unique, productive research and teaching relationships.

**Blue Sky Ideas for Solving This Challenge:**

Far-fetched ideas for solving current challenges in research and implementation of predictive analytics commonly focused on promoting large, collaborative science. These ideas include reimagining funding models to promote inclusivity of those outside the fields of plant and animal sciences. One such idea is to create a reward system similar to the X-prize where research teams focus on clear targets or measurable goals and develop the tools and methodologies to accomplish these goals. X-prize competitions have been successful in bringing diverse individuals together to imagine and develop unique solutions. Additional ideas included hosting hackathons to develop, test, and identify the best predictor tools and software. These hackathons would be open to all individuals regardless of area of expertise or domain.

**References:**

Abbot, D., 2014. Applied predictive analytics: Principles and techniques for the professional data analyst. John Wiley & Sons. (ISBN: 978-1-118-72796-6)

**Advancing Genomic Research**

Ensuring food security in the face of changing climate necessitates the advancement of genomic research. Thus, the entire spectrum of genomics research, ranging from sequence and structural genomic variation to the application of this information to breeding programs, need to be critically evaluated. Recent improvements in production levels of agricultural species have been driven by the availability of high-quality genomes and related -omics datasets. These genomes have accelerated the use of genomic selection, as well as other revolutionary analyses, due to unprecedented identification of genomic variants. However, as we discussed the challenges of advancing genomic research, it has become clear that more data and more tools are necessary. The manifestation of this need comprises identification of variants such as indels and copy number variations, derived from sequencing additional individuals from within a species and more broadly across species, as well as epigenomic variation, derived from methyl-seq, ChIP-seq, and ATAC seq. This improved knowledge will drive even more application of genomic technology in breeding. In addition, we identified non-scientific hurdles that are discussed below.

There is a general misconception that the issue of genomics is “solved” for any given species because only a few (or in some scenarios, only one) genomes have been sequenced. While the sequencing of agricultural genomes was largely initiated in the early 2000’s, many of these genomes were derived from individuals that were inbred to some extent. These individuals were chosen to facilitate genome assembly and save costs. However, extensive sequencing of these individuals also reduced the extent of discoverable genetic diversity and were not representative of commercial genomes. Consequences of this are now evident as we try to dissect the genetic architecture of economically important traits in plants and animals. Fortunately, the cost of genomic sequencing has decreased over the past two decades as sequencing methods and bioinformatic tools have improved. To adequately utilize the genomic contribution from high-throughput phenotyping to novel trait information being obtained, more individuals need to be sequenced at both genomic and epigenomics levels. Importantly, this should include rare and endangered breeds/landraces with unique phenotypes, as well as more relevant species (e.g., wild progenitors).

Insufficient translation of advancements in basic genomic research to practical, usable analytical tools is particularly dangerous as it will inhibit optimal plant and animal germplasm development. Consequently, the full potential for genomic selection to accelerate genetic gain in agronomically important traits and species will become unrealized, especially if we are unable to account for critical sources of genomic variation using currently available tools and data. Unprecedented gains in milk yield in dairy cattle are a direct example of the success of genomic selection. Without additional genomic advances, successes like this will not be realized in other agronomically important species. To prevent this from happening, there is a critical need to genotype and/or sequence individual samples that are representative of the genomic diversity of a species as a whole; i.e., a pan-genome. Otherwise, genomic estimated breeding values of selected germplasm could become unrepresentative of the actual phenotypic values breeders might expect to observe in real life.

The challenges inhibiting our advancement of genomic research are predominantly non-scientific. Administrative burdens resulting from loss of staff mean that scientists have less research time. As public support for genomics wanes, support staff are cut, and research-focused scientists must spend more time on non-science responsibilities such as teaching, human resources, management, oversight, and administration. Coupled with decreased available funding and a smaller pool of trained scientists, the genomics community is struggling to figure out how to continue to be productive in the face of these additional responsibilities. The biggest challenge from a scientific perspective is to ensure that there is continued support of a basic pillar of scientific research: that knowledge is gained from failures and incorporated into better experiments and breeding protocols in the future.

Solutions to overcome these challenges were subdivided into short-, medium-, and long-term strategies. The immediate next step for the agricultural genomics community is to make higher-resolution, more diverse genomic data available. In particular, there is a critical need for more pan-genomes and pan-epigenomes. Concurrently, statistical and visual tools that are truly accessible to all are urgently needed, along with training on how to use and access these tools. This training is essential because all tools and data need to be useable, easily transferable, and understandable to a broad audience. For the medium term, efforts need to be taken to identify, quantify and characterize a wider variety of variants besides SNPs, as other variants (e.g., CNVs) and genomic markers (e.g., DNA methylation) also impact phenotypes. These variants need to be identified and made available in a wider variety of i.) individuals within species and ii.) across relevant populations of domesticated species and their wild progenitors. The long-term goals are to have all of the resulting variants and statistical/visualization tools made available using formatting standards that are widely accepted across the spectrum of genomics research. While we have discussed these solutions with both plants and animals in mind, we realize that having a more cohesive agricultural genomics community will allow us to learn from each other and save time by solving common problems together.

Finally, blue-sky ideas were discussed for solving challenges in the more distant future. These ideas were the most dissimilar from other topics discussed and were the most multifaceted. The most prominent scientific blue-sky ideas centered around single-cell sequencing. Specifically, we talked about initiating an absolutely mind-blowing and nimble single-cell resolution genomics project across a large number of species, resulting in a program that is at the scale and cost of the original human genome project. The resulting data would provide functional insight into cellular heterogeneity and enable the genomic selection of complex traits to become highly refined with respect to cell type and cellular response to environmental conditions.

This would advance the underlying technology to generate data as well as the methods for analyzing and managing them.

In conclusion, it was widely agreed that the perception of genomics being “done” is a prevalent misconception that will prevent us from developing the tools and resources needed to tackle future challenges. It is imperative that we invest in generating a wide variety of sequences for a wide variety of individuals within and across species (both domesticated and feral populations). Moreover, we need to bolster education and funding efforts to regenerate enthusiasm for advancing genomics research. Otherwise, the scientific community may become ill-equipped to advance genetic gain in a timely manner.
