# Supplementary material for: Current challenges and future of agricultural genomes to phenomes in the USA
Source: Genome Biol. 2024 Jan 3;25:8. doi: 10.1186/s13059-023-03155-w (PMC10763150; doi:10.1186/s13059-023-03155-w)
Supplement: Supplementary file 3 — Additional file 3: Review history. [file 13059_2023_3155_MOESM3_ESM.docx]

**1^st^ round**

**Reviewer 1**

This paper has been written to summarize the outcomes of a workshop between crop and livestock researcher, spcial scientists, data scientists, engineers and stakeholders that had the aim to envision the future of the Agriculture Phenome to Genome Initiative (AG2PI). Several challenges were discussed and possible solutions proposed during this workshop. This workshop/paper concludes that the two main areas where innovation is needed are genetic improvement methods and agricultural research processes to solve societal problems. The paper proposes six potential research topics that should be prioritized to support AG2PI research. The paper is a follow-up to the AG2PI white paper published two years ago by a subset of the authors (https://doi.org/10.1186/s13059-021-02570-1).
While this is generally a useful and well-written report, I have to admit that I don't find the paper to present terribly novel insights or recommendations (possible solutions include better education, data sharing, inter- and transdisciplinarity).
It's likely the format of the paper / workshop, but it may be worth to put this very U.S.-centric view better into global context.

**Authors’ response**

Editors: As you will see, Referee 1 asks that you try and add a bit of global perspective.

Authors: We revised several passages and added some sentences to address this point.
